# From tragedy to triumph: Rwanda’s healthcare over three decades

**DOI:** 10.1097/MS9.0000000000003237

**Published:** 2025-07-08

**Authors:** Olivier Uwishema, Abdelmonem Siddiq, Jack Wellington, Sarah Mshaymesh

**Affiliations:** a Oli Health Magazine Organization, Research and Education, Kigali, Rwanda; b Faculty of Pharmacy, Mansoura University, Mansoura, Egypt; c Bradford Teaching Hospitals NHS Foundation Trust, Bradford, UK; d Division of Natural Sciences, Faculty of Sciences, Haigazian University, Beirut, Lebnon

Thirty-one years ago, Rwandans were confronted with the despicable mass genocide against the Tutsi in 1994. This had precipitated vast extremes in mortality rates, as well as collapse of one of the most critical national sectors in the country – its healthcare system.

Essentially, the healthcare system of Rwanda had been successful in large part due to the Community-Based Health Insurance (CBHI) administration, colloquially referred to as “Mutuelle de santé.” This comprehensive insurance programme has ensured that Rwandans deemed impoverished may still obtain healthcare services despite their cumbersome financial difficulties. In addition, it provided access to affordable treatments and promoted economic growth through long-term wellbeing^[1,2]^. CBHI consists of electing three healthcare workers by each village’s members, which are then trained and equipped to deliver healthcare services. This has enhanced the financial status in Rwanda, as well as the quality of service, since it relies on new technological platforms for better monitoring. Many services are fully covered by CBHI, such as vaccinations against the commonest infections in the region, and these services are mainly focused on child and maternal health^[2,3]^.

Furthermore, the introduction of the Community Health Worker (CHW) program of 1995 post-Tutsi genocide has played a substantial role in the revival of Rwandan medical care. This offered many resources deemed fit for purpose, comprising antimicrobial chemotherapeutic agents against malaria and tuberculosis, further malnutrition assessment tools, as well as contraceptives and mother-and-child care. Their contribution to Rwanda’s healthcare sector goals and subsequent progress towards the Millennium Development Goals has been vital^[2-4]^.

Moreover, increasing the number of healthcare practitioners working in Rwanda was notable. Following the implementation of several strategic investments in medical education and training, Rwanda has fortified its healthcare system with the objective of meeting the requirements of its people. Additionally, collaborations with foreign governmental and non-governmental organizations, including Partners in Health (PIH) founded by the late Professor Doctor Paul Farmer, have been crucial in providing Rwandan healthcare institutions with assistance, professionalism, and knowledge in its pursuit for the betterment of patient-centered care as shown in Fig. 1. and in Table 1^[1,4-7]^. All these advances in Rwanda’s healthcare system would be very important in emergencies, such as a very recent outbreak of the first cases of Marburg virus in Rwanda^[7]^. These facilities will allow Rwanda to collaborate with international institutions and prepare a management plan of the disease^[7]^.

**Figure 1.**
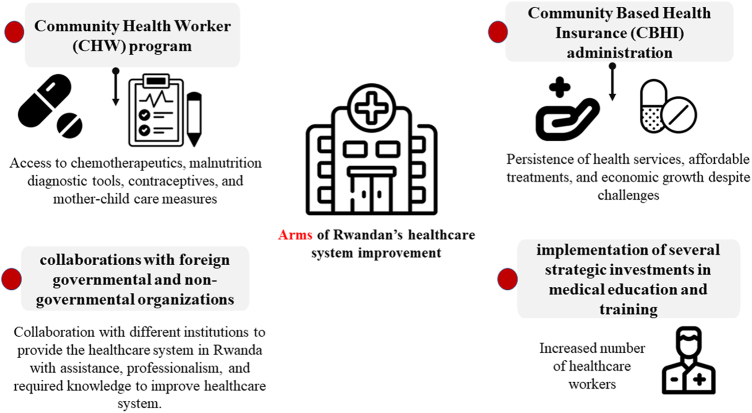
Different measures that helped Rwanda to improve its healthcare system.

**Table 1:** Table summarizing key healthcare reforms and outcomes in Rwanda in each study

Author (s)	Study type & journal	Key findings
Uwishema et al. (2023)	Correspondence *(The Lancet)*	Availability of healthcare services despite financial difficulties
Binagwaho et al. (2014)	Review *(The Lancet)*	Community Based Health Insurance (CBHI) and Community Health Worker (CHW)
		Many resources supplementation for malaria and tuberculosis
Nsanzimana et al. (2015)	Review *(BMC Medicine)*	Antimicrobial agents for malaria and tuberculosis
Farmer et al. (2013)	Review (*BMJ)*	
		dvancement in educational programs
		Patient-centered care
Schmidt et al. (2013)	Case study analysis *(The Yale Journal of Biology and Medicine)*	Increased number of healthcare practitioners
		Medical education and training
		Collaboration with organisations
Cancedda et al. (2018)	Retrospective observational (*International journal of*	
	*health policy and management)*	
Uwishema et al. (2024)	Correspondence *(The Lancet)*	Importance of preparedness of Rwanda for emergencies, such as the Marburg virus outbreak.

Since Rwanda is now confronting other limitations in delivering health maintenance, it is imperative to confess the accumulated experience and pursue building on previous successes. By prioritizing equality and modernization alongside employing collectivist ideologies, Rwanda has the potential to maintain its position as a worldwide leader in healthcare systems and serve as an example for other countries who are unable to offer their population access to healthcare resources.

## Data Availability

Not Applicable.

## Research Registration Unique Identifying Number (UIN)

Name of the registry: **Not Applicable**, Unique Identifying number or registration ID: **Not Applicable**, Hyperlink to your specific registration (must be publicly accessible and will be checked): Not Applicable.
